# Non-Gaussian tail in the force distribution: a hallmark of correlated disorder in the host media of elastic objects

**DOI:** 10.1038/s41598-020-76529-w

**Published:** 2020-11-10

**Authors:** Jazmín Aragón Sánchez, Gonzalo Rumi, Raúl Cortés Maldonado, Néstor René Cejas Bolecek, Joaquín Puig, Pablo Pedrazzini, Gladys Nieva, Moira I. Dolz, Marcin Konczykowski, Cornelis J. van der Beek, Alejandro B. Kolton, Yanina Fasano

**Affiliations:** 1 Centro Atómico Bariloche and Instituto Balseiro, CNEA, CONICET and Universidad Nacional de Cuyo, 8400 San Carlos de Bariloche, Argentina; 2 Universidad Nacional de San Luis and Instituto de Física Aplicada, CONICET, 5700 San Luis, Argentina; 3 Laboratoire des Solides Irradiés, CEA/DRF/IRAMIS, Ecole Polytechnique, CNRS, Institut Polytechnique de Paris, 91128 Palaiseau, France; 4 Centre de Nanosciences et de Nanotechnologies, CNRS, Université Paris-Saclay, 91120 Palaiseau, France

**Keywords:** Materials science, Physics

## Abstract

Inferring the nature of disorder in the media where elastic objects are nucleated is of crucial importance for many applications but remains a challenging basic-science problem. Here we propose a method to discern whether weak-point or strong-correlated disorder dominates based on characterizing the distribution of the interaction forces between objects mapped in large fields-of-view. We illustrate our proposal with the case-study system of vortex structures nucleated in type-II superconductors with different pinning landscapes. Interaction force distributions are computed from individual vortex positions imaged in thousands-vortices fields-of-view in a two-orders-of-magnitude-wide vortex-density range. Vortex structures nucleated in point-disordered media present Gaussian distributions of the interaction force components. In contrast, if the media have dilute and randomly-distributed correlated disorder, these distributions present non-Gaussian algebraically-decaying tails for large force magnitudes. We propose that detecting this deviation from the Gaussian behavior is a fingerprint of strong disorder, in our case originated from a dilute distribution of correlated pinning centers.

## Introduction

Elastic objects nucleated and driven in disordered media represent an ubiquitous situation in nature, covering diverse fields of research such as defect and crack nucleation and propagation in materials^1,2^, domain wall dynamics^3–5^, charge density waves^3,6^, photonic solids^7^, randomly-packed objects^8^, magnetic bubbles nucleated in substrates^9^, colloidal spheres^10^, to avalanches in magnets^11^ and vortex matter in superconductors^12–14^. The physical properties of these systems with different types of particle-particle interaction has been the subject of a wide and interdisciplinary field of research^3–20^. Knowledge on the nature of disorder in the host media is of crucial importance for many applications but in most cases requires potentially destructive micro-structural characterization. Inferring the nature of disorder via a non-destructive approach relying on information from physical properties of the elastic objects remains an open problem.

Research on vortex matter nucleated in type-II superconductors has shed light on the structural properties of elastic objects nucleated in media with different types of disorder^21–47^. A handful of works study the spatial distribution of particle-particle interaction forces to get information on the vortex-disorder interaction^48–54^. Superconducting vortices are repulsively-interacting elastic lines but, due to the pressure exerted by the applied field, they tend to form a hexagonal lattice with a spacing $a_{0}$ tuned by the magnetic induction *B*, namely $a_{0} \propto B^{-1/2}$. In addition, vortices are pinned by disorder, structural defects naturally present or introduced in the host superconducting samples. As in many systems of interacting elastic objects, the structural properties of vortex matter result from the balance between thermal, particle-particle and particle-disorder interaction energies. Many theoretical and experimental studies describe the structural deviations from a perfect hexagonal vortex lattice induced by different pinning landscapes^12,21–47,55–59^. Correlation function and structure factor studies have been performed in order to characterize the structure of the different glassy phases stabilized by disorder with different geometrical properties, including randomly-distributed point pins^43,45,46^ and correlated defects^21,22,53^, as well as periodic distributions of pinning sites^17^. Recently, some studies focusing on characterizing long^46,60^ or short-range^47^ vortex density fluctuations obtained contrasting results for samples with point or correlated disorder, see the discussion in Supplementary Note 1. From the perspective of technological applications, having information on the nature and distribution of disorder in a given sample is crucial since correlated in addition to point-like disorder is more efficient to pin vortices and thus to enhance the material critical current^12^.

With the aim of inferring the nature of disorder in the host medium from physical properties of the elastic objects, in this work we follow a novel approach and study the inhomogeneous spatial distribution of the particle-particle interaction force in extended fields-of-view. Previous works by some of us combined the study of interaction energy and force maps to infer information on the typical separation between strong point disorder in pnictide superconductors^48,50,51^. But here we go beyond that and study the more general problem of discerning whether the host medium presents point or correlated disorder. In this work, our case-study elastic system is the vortex matter nucleated in the layered high-$T_{\text{c}}$ superconductor $\hbox {Bi}_2\hbox {Sr}_2\hbox {CaCu}_2\hbox {O}_{8+\delta }$. We study samples with different types of pinning landscapes representative of different classes of randomly distributed disorder: Naturally occurring weak and dense *point* pins in pristine samples, extra moderate and dense *point* pins generated by electron irradiation, and columnar-defects (CD) responsible for *correlated* pinning. In the latter case, the pinning centers are columns of crystallographic defects generated via heavy-ion irradiation, that traverse the whole sample thickness and are distributed at random in the plane perpendicular to the direction of vortices. We study samples with dilute and dense distributions of correlated disorder quantified by the matching field $B_{\Phi }=N_{CD} \cdot \Phi _{0}$ proportional to the number of CD per unit area, $N_{CD}$, and the magnetic flux quantum $\Phi _{0}=2.07 \cdot 10^{-7}$ $\hbox {G}\,\cdot \,\hbox {cm}^2$. Our samples with diluted CD disorder have $B_{\Phi }= 30, 45$ and 100 G, whereas a sample with a larger $B_{\Phi }= 5000$ G is also studied. Then, this work presents a comprehensive corpus of data in $\hbox {Bi}_2\hbox {Sr}_2\hbox {CaCu}_2\hbox {O}_{8+\delta }$ samples with a diversity of pinning centers with different nature and spatial density.

The novel analysis presented in this paper, in contrast to previous works, focus on the study of the tails in the probability density function of the components of the vortex–vortex interaction force. This method, based on imaging of vortex positions, gives new insight on the nature of disorder in the host media. In addition, it presents advantages over previous studies on mean or typical values of several properties since the statistics of rare events appear to be particularly sensitive to the nature of disorder, point or correlated. Furthermore, the occurrence of non-Gaussian fluctuations is per se a subject of broad interest in several fields of Physics such as galaxy clustering, critical points in phase transitions, anomalous diffusion, Econophysics, out of equilibrium and active systems, to name just a few. The experimental realization of the method presented here has also the advantage of, although being invasive at the top layer of the sample, not being destructive. Of course other less invasive imaging techniques such as magnetic force microscopy^47^, scanning tunnelling microscopy^43,60^, scanning squid microscopy^61^, or scanning Hall probe microscopy^62^ would be suitable to perform this analysis as well. Nevertheless, the magnetic decoration technique has the advantage over the mentioned techniques of imaging with single vortex resolution in very large fields-of-view with thousands of vortices, allowing for studies with larger statistics.

## Results

**Figure 1:**
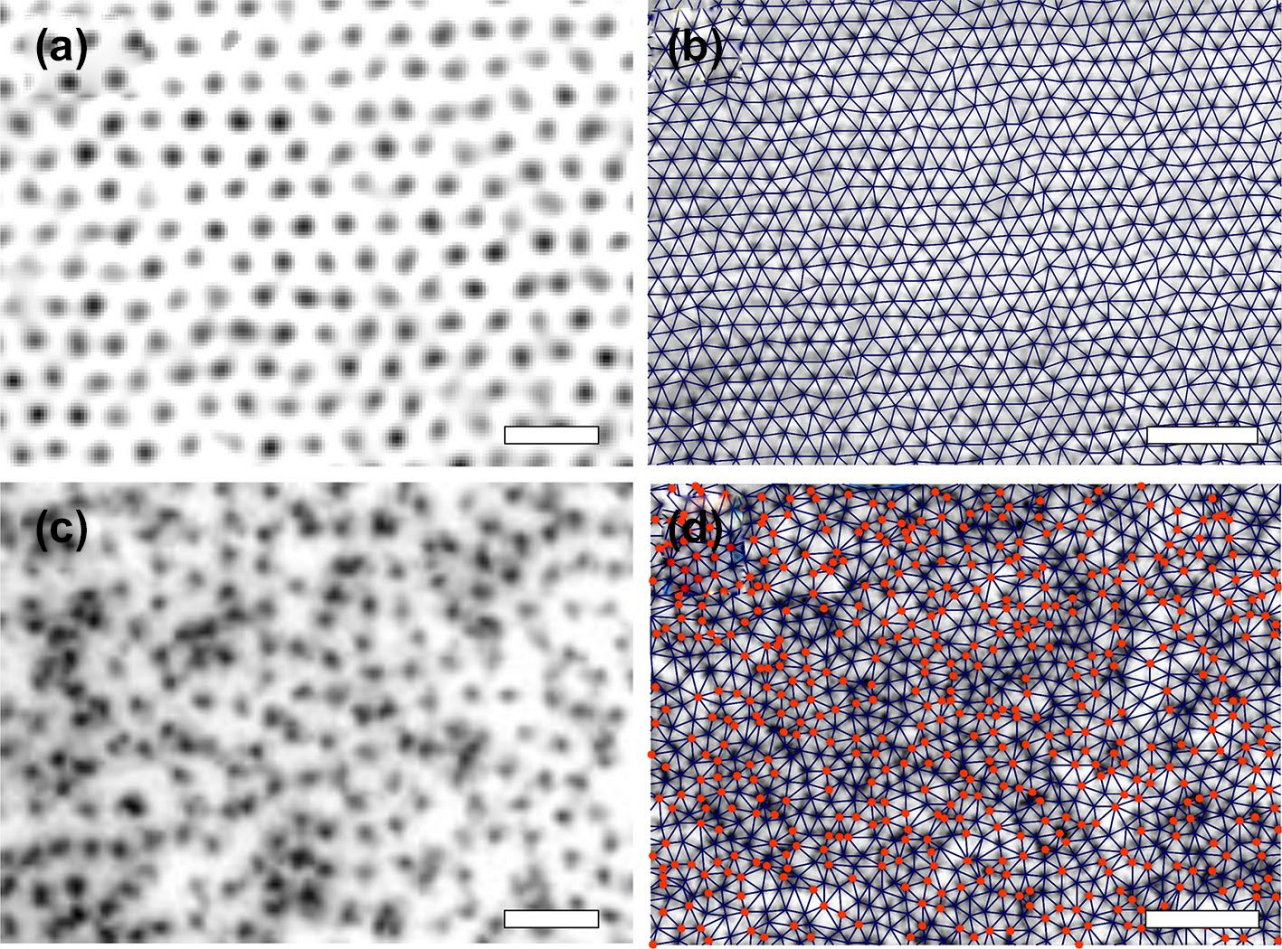
Magnetic decoration snapshots of vortices (black dots) nucleated at 30 G in $\hbox {Bi}_2\hbox {Sr}_2\hbox {CaCu}_2\hbox {O}_{8+\delta }$ samples: (**a**, **b**) Pristine sample with weak point disorder; (**c**, **d**) sample with correlated disorder generated by a diluted density of columnar defects with matching field $B_{\Phi }=30$ G. Left images cover a $17\,\times \,15$ $\mu \hbox {m}^{2}$ field-of-view with the white bar corresponding to 2 $\mu \hbox {m}$; right images also cover a larger field-of-view with the white bar corresponding to 5 $\mu \hbox {m}$. The right panels show superimposed Delaunay triangulations, an algorithm that identifies first-neighbors, here joined with blue lines. Highlighted in red are non-sixfold coordinated vortices, i.e. topological defects in the hexagonal structure. These images are zoom-ins of larger snapshots with approximately 7000 vortices.

In the ideal case of mechanical equilibrium, for a static configuration of vortices at a fixed temperature, the vortex–vortex (i.e. the particle–particle) interaction force is compensated by the vortex–pinning interaction. Therefore, the spatial distribution of particle–particle interaction force allows for the estimation of local pinning forces at the temperature at which the snapshot was captured^48–54^. In our experiments we obtain these snapshots by decorating vortex positions with magnetic nanoparticles after following a field-cooling procedure (see “Methods” for technical details). Even though the snapshots are taken at 4.2 K, the vortex structure observed by means of magnetic decoration is frozen at temperature $T_{{\text{freez}}}>>4.2$ K given by disorder inhibiting vortex motion at lengthscales larger than the vortex spacing^53,63^. Once $T_{{\text{freez}}}$ for a given host medium is determined (see the Supplementary Note 2 for details on how we measure the field-evolution of $T_{{\text{freez}}}$), the particle-particle interaction force per unit length for a given vortex *i* with the rest of the *j*-th vortices of the structure can be computed as^12^

1 $$\begin{aligned} {\mathbf {f}}_{\text{i}}({\mathbf {r_{i}}})=\frac{2\epsilon _0}{\lambda (T_{{\text{freez}}})} \sum _{j} \frac{{\mathbf {r}}_{\text{ij}}}{r_{\text{ij}}} K_{1}\left( \frac{r_{\text{ij}}}{\lambda (T_{\text{freez}})}\right) \end{aligned}$$

This expression is valid for superconductors with large $\kappa =\lambda /\xi$ values as $\hbox {Bi}_2\hbox {Sr}_2\hbox {CaCu}_2\hbox {O}_{8 +\delta }$, with $\lambda$ the in-plane penetration depth and $\xi$ the coherence length, and in the low vortex density range $a_{0} \gg \lambda$ covered in our experiments. The magnitudes in Eq. (1) stand for: ${\mathbf {r}}_{\text{i}}$ the location of vortex *i*; ${\mathbf {r}}_{\text{ij}}$ the vector separation between vortices *i* and *j*; $\epsilon _{0}= (\Phi _{0}/4\pi \lambda _{\text{ab}}(T_{\text{freez}}))^2$ an energy scale proportional to the vortex line energy and $K_{1}$ the first-order modified Bessel function^12^. The sum runs for all vortices in the sample but, for the low-density vortex structures studied here, the contribution from vortices separated more than $\sim 10 a_{0}$ is negligible.

Maps of ${\mathbf {f}}_{\text{i}}({\mathbf {r_{i}}})$ depicting its spatial variation can be obtained from digitalizing vortex positions in magnetic decoration snapshots as those shown in Fig. 1. These images correspond to zooms on structures of $\sim 7000$ vortices nucleated at 30 Gauss in media with point (top panels) and correlated CD disorder (bottom panels). In the former case the vortex structure has long-range orientational hexagonal order and there are no apparent short-scale density fluctuations. In contrast, the vortex structure nucleated in the medium with correlated disorder presents noticeable degradation of the hexagonal symmetry and strong short-scale vortex density fluctuations, with a tendency to clustering at some particular locations. Nevertheless, even if the density of vortices in Figs. 1c and d is equal to the global density of randomly distributed CD ($B_{\Phi }=30$ G), not every vortex is located on top of a correlated defect. This last assertion is proved in detail in the Supplementary Note 3. Thus, the vortex clustering observed in samples with dilute correlated disorder is connected to the spatial distribution of CD’s, but the structure imaged in the entire field-of-view does not mimic the random Poissonian distribution of these correlated pins. Therefore, characterizing the spatial distribution of vortices at densities $B/B_{\Phi }=1$ is not an unambiguous way to ascertain whether the dominant disorder in the medium is point or correlated.

This contrast between the short-scale density-variation of vortex structures nucleated in point- and correlated-disordered media is systematically found in larger field-of-view images, irrespective of the vortex density. Indeed, at a given vortex density, the spatially inhomogeneous distribution of first-neighbor distances *a*, is larger for dilute correlated than for point disorder. The Supplementary Note 4 presents the magnetic field dependence of the standard deviation *SD* of *a* normalized by the mean lattice spacing $a_{0}$ for all studied samples. As discussed there, despite this quantitative difference in $SD/a_{0}$, the value and field-evolution of this magnitude is not a qualitative indicator of disorder being point or correlated in nature. At best, values of $SD/a_{0}>0.2$ might be taken as a hint that in this particular vortex system the medium presents dilute correlated random disorder.

The panels (b) and (d) of Fig. 1 also present superimposed Delaunay triangulations that join first-neighbors with blue lines^17^. Topological defects formed by non-sixfold coordinated vortices are highlighted in red. These images are representative of results found in larger fields-of-view. An analysis on the density of non-sixfold coordinated vortices, $\rho _{\text{def}}$, in each type of medium reveals that for this vortex density the structure nucleated in point disorder is single-crystalline whereas the one nucleated in correlated disorder is amorphous. However, at smaller vortex densities the $\rho _{\text{def}}$ enhances and even reaches similar values in structures nucleated in point- and correlated-disordered media, see Supplementary Note 4 for details. Then the value of $\rho _{\text{def}}$ is neither a good candidate for distinguishing between point and correlated disorder.

**Figure 2:**
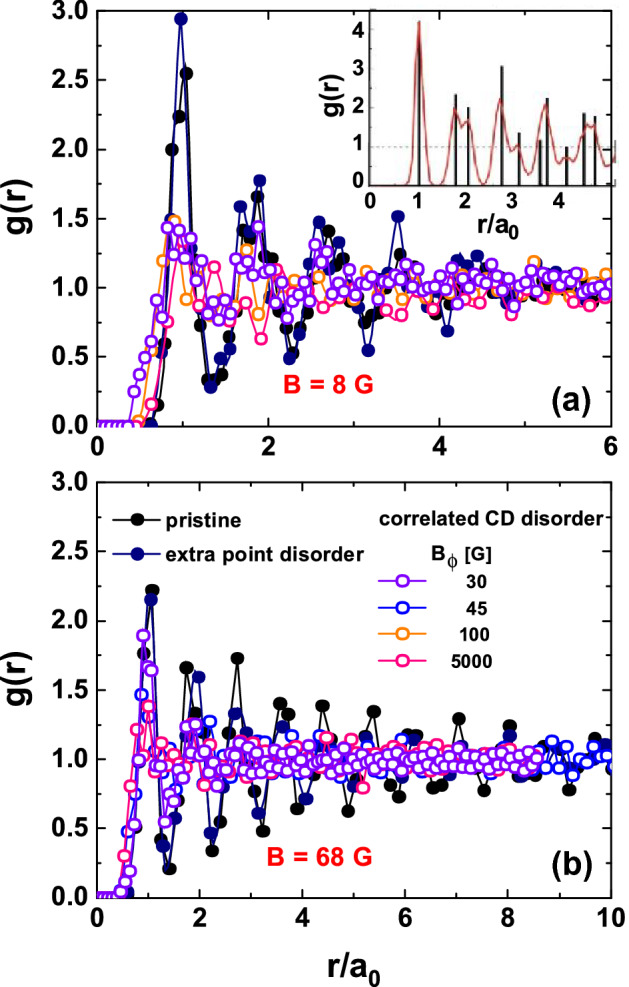
Pair correlation functions of vortex structures nucleated in $\hbox {Bi}_2\hbox {Sr}_2\hbox {CaCu}_2\hbox {O}_{8+\delta }$ samples with point (pristine and electron irradiated) and CD correlated (heavy-ion irradiated) disorder. Results at two different vortex densities of (**a**) 8 and (**b**) 68 G. The legend shows the color-code used for the different medium studied in both panels. Inset at the top: pair correlation function for a perfect hexagonal structure (black delta functions) and widening of the peaks due to random fluctuations around the sites of a perfect lattice (red curve).

**Figure 3:**
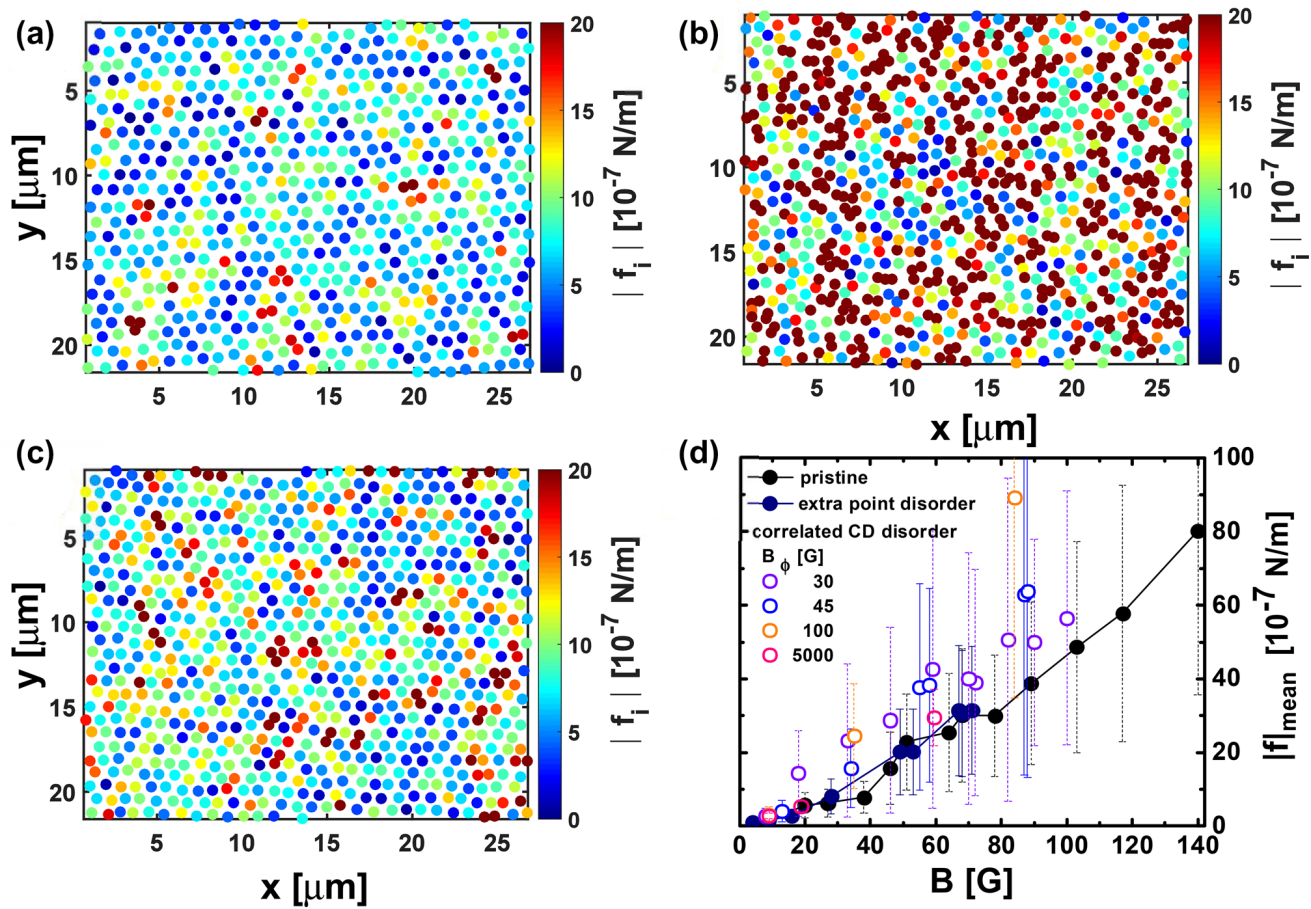
Colour-coded maps of the modulus of the particle-particle interaction force $|f_\text{i}|$ for 30 G vortex structures nucleated in $\hbox {Bi}_2\hbox {Sr}_2\hbox {CaCu}_2\hbox {O}_{8+\delta }$ samples with point ((**a**) pristine, (**c**) electron- irradiated) and correlated CD disorder ((**b**) heavy-ion irradiated with $B_{\Phi }=30$ G). (**d**) Density (field)-dependence of the mean value of $|f_\text{i}|$, $|f|_{{\text{mean}}}$, obtained from large field-of-view maps with 7000-15000 vortices for all the studied structures. Bars accompanying the points are not error bars but the value of the standard deviation of the $|f_\text{i}|$ distribution registered in such extended fields-of-view.

Another possible path to reach this ascertainment might be to study the pair correlation function *g*(*r*) at different particle-to-pinning sites density-ratio. This function describes how the density of particles varies as a function of distance from a reference point. For a structure with mean lattice spacing $a_0$, the *g*(*r*) quantifies the probability of finding a particle at a given distance $r/a_0$ from the origin, averaging this probability when considering every particle of the structure at the origin. This is therefore an angular-averaged probability that gives information on short- and intermediate-distance vortex density variations since $g(r) \rightarrow 1$ for *r* larger than some units of $a_{0}$. Figure 2 shows the *g*(*r*) for vortex structures nucleated at two different vortex densities of 8 and 68 G, in three classes of disordered medium. These data will be relevant for the discussion presented in the next section, but as we discuss in the Supplementary Note 5, studying the shape of *g*(*r*) for $r/a_{0} >1$ is not a promising way to determine whether disorder in the medium is dominated by point or correlated pins.

We pursue our search of an unambiguous indicator in the physical properties of the elastic structure for distinguishing correlated from point disorder in the medium, by finding a magnitude that should have a qualitatively different behavior for both types of disorder. This property might be a particular feature of the statistic distribution of the locally-varying particle–particle interaction force that entails information on the short-scale density fluctuations induced by disorder in the media. With this aim we map the vortex–vortex interaction force ${\mathbf {f}}_{\text{i}}({\mathbf {r_{i}}})$ in extended fields-of-view following the expression of Eq. (1). Maps of the modulus of the local force, $|f_{\text{i}}|$, are shown in Fig. 3 for the structures presented in Fig. 1, and also for vortex lattices nucleated in samples with extra point disorder. In all cases the vortex density corresponds to $B=30$ G. There is no noticeable spatial pattern in the $|f_{\text{i}}|$ maps of vortex structures nucleated in samples with point disorder, see panels (a) and (c). On the other hand, clusters of bordeaux vortices with larger modulus of the interaction force are distinguished in the case of the correlated disordered medium, see panel (b). These regions correspond to areas in which the vortices are closer than in the rest of the structure, presumably induced by a locally denser concentration of CD distributed at random in the sample. The mean of the local values of $|f_{\text{i}}|$, $|f|_{\text{mean}}$, is plotted in Fig. 3 (d) as a function of field and for all the studied media. The values of $|f|_{\text{mean}}$, a reasonable estimate of the average pinning force^48,51^, are in the range $10^{6}\,-\,10^{5}$ Nw/m in the whole field range. This magnitude is always larger in vortex structures nucleated in samples with strong correlated than with weak point disorder: Globally between 30 to 50 % larger, and at high fields even reach a value 300 % larger for the particular sample with $B_{\Phi }=100$ G. The case of a medium with a dense distribution of CD ( $B_{\Phi }=5000$ G sample) is rather special since the $|f|_{\text{mean}}$ values are close to those of samples with point disorder at low fields. However, at large *B* the $|f|_{\text{mean}}$ curve for the $B_{\Phi }=5000$ G sample enhances significantly and tends towards the values found in the case of dilute correlated disorder. Then, due to their relative quantitative nature, this global magnitude is not appropriate to ascertain the degree of correlation of the pinning sites.

**Figure 4:**
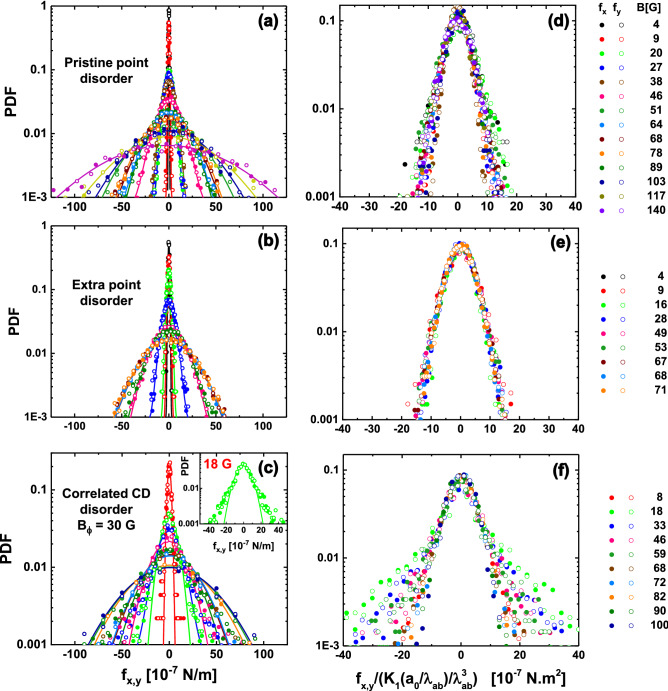
Probability density functions (PDF’s) of the particle-particle interaction force components $f_x$ (full points) and $f_y$ (open points) for vortex structures nucleated at various applied fields in $\hbox {Bi}_2\hbox {Sr}_2\hbox {CaCu}_2\hbox {O}_{8+\delta }$ samples with point (**a**) pristine, (**b**) electron irradiated, and (**c**) correlated CD disorder with $B_{\Phi }=30$ G. Data are presented in log-linear scale. Full lines are fits to the data with a Gaussian function $\propto (1/\sigma _\text{G}) \cdot \exp (-x^2/2\sigma _\text{G}^2)$ in the whole force range. This function describe reasonably well the PDF’s in the case of point disorder, but in the case of correlated disorder fail to follow the experimentally-observed tails that decay slowly with $f_{x,y}$ than a Gaussian function. The inset in (**c**) shows the example of the 18 G data where it is evident that the Gaussian function (full green line) underestimates the experimental data in the large-force range. The right panels (**d**–**f**) show the PDF’s of the left panels plot in log-linear scale with the *x*-axis normalized by the factor $K_{1}(a_{0}/\lambda _{\text{ab}})/\lambda ^{3}$ and then normalized to have an area under the curve equal to one. In this representation, for point disorder the PDF’s scale in a single evolution whereas for dilute correlated disorder the curves do not overlap in the large-force range.

To grasp on this issue, we rely on magnitudes that significantly depend on the local variations of vortex density that are presumably larger and more inhomogeneous in the case of correlated disorder. These magnitudes are the in-plane components of the interaction force, $f_{x}$ and $f_{y}$, that we also map in extended fields-of-view. Examples of the probability density functions (PDF’s) for both components are shown in Fig. 4 for structures nucleated in samples with point and dilute correlated CD disorder ($B_{\Phi }=30\,\hbox {G}$ in this case). These data are some examples of the more than 50 cases studied covering vortex densities between 4 and 140 G and samples that are pristine, that have extra point and dilute or dense correlated CD disorder. In all our experimental data, the mode values of the PDF’s of $|f_{\text{i}}|$ are finite (since the observed structures are far from being a perfect hexagonal one), but the mode values of the PDF’s of the force components are equal to zero since the positive and negative *x* and *y* directions of space are equivalent. Higher values of the components of the particle-particle force enhance their probability of occurrence on increasing *B* since vortices get closer to each other, irrespective of the type of disorder of the host medium.

Nevertheless, the right panels of Fig. 4 show a scaling of the data that highlight that the shape of the PDF for large $f_{x,y}$ values is different for point than for dilute correlated disorder. This scaling is made by dividing the force components by a factor $K_{1}(a_{0}/\lambda (T_{\text{freez}}))/\lambda (T_{\text{freez}})^{3}$ proportional to the average interaction force for each studied field in a given material. While for point disorder all curves collapse in a single trend, that is not the case for dilute correlated disorder in the large-force range. In the latter case, the tails of the scaled distributions do not overlap and become narrower on increasing field, see Fig. 4f. Going back to the non-scaled data shown in the left panels, the PDF’s for structures nucleated in a medium with point disorder fit a Gaussian distribution function $(1/\sigma _{\text{G}})\cdot \exp (-x^2/2\sigma _{\text{G}}^2)$, see full lines in (a) and (b). In contrast, Fig. 4c shows that for dilute correlated CD disorder a fit to the PDF’s with a Gaussian function (for the whole force range) only follows the experimental data in the low-force range. In the large-force range, the experimental data decay at a slower pace and non-Gaussian tails are observed. This is clearly observed in the example shown in the inset to this figure for the 18 G vortex density. These non-Gaussian tails become wider at low vortex densities, i.e. the Gaussian fit is gradually underestimating the experimental data on decreasing *B*. This finding is also observed in the scaled-PDF’s of Fig. 4f.

**Figure 5:**
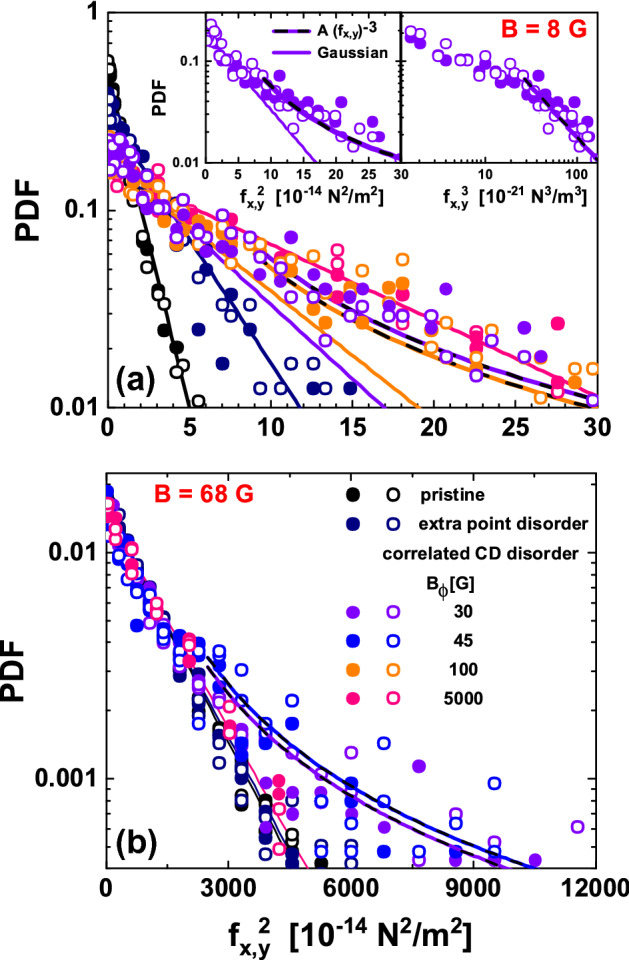
Probability density functions (PDF’s) of the vortex-vortex interaction force components as a function of $f_{x}^2$ (full points) and $f_{y}^2$ (open points) for densities of (**a**) 8 and (**b**) 68 G for structures nucleated in samples with point and correlated disorder with different CD densities. Fits to the data with Gaussian functions (full lines) and with an algebraic decay $\propto f_{x,y}^{-3}$ (black-dashed on top of color lines) are presented. Insets: Detail of the 8 G structure nucleated in a sample with correlated disorder ($B_{\Phi }=30$ G). Left: PDF data plotted as a function of $f_{x,y}^2$ with Gaussian fit in full line and algebraic fit in black-dashed on top of color line. Right: Data plotted as a function of $f_{x,y}^3$ and the same algebraic function shown in the left inset.

Figure 5 shows a different representation of some examples of the studied cases including data in samples with point and correlated CD disorder with different $B_{\Phi }$, both dilute and dense. Panel (a) of this figure shows PDF data at a density of 8 G and panel (b) shows data at 68 G for all the studied media. The data are plotted in a log-linear scale as a function of $f_{x,y}^{2}$, a representation that puts in evidence that when the host media present point disorder, the PDF’s of the force components follow a Gaussian distribution, irrespective of the vortex density. The same figure shows that for a medium with diluted CD disorder the PDF’s of the force components follow a Gaussian function only in the small-force range. However, in the range of intermediate- and large-force components, the PDF’s plotted as a function of $f_{x,y}^{2}$ in log-linear scale do not follow the apparent linear behavior. This is clearly illustrated in the left inset to Fig. 5a showing the example of the 8 G data for a sample with $B_{\Phi }= 30$ G: Data departs from linearity (full line) at intermediate values of the force components and its decay is slower within the large-force range. In this force-range where the non-Gaussian tails develop, the PDF’s are well described by the algebraic decay $\propto f_{x,y}^{-3}$, see for instance the apparent straight-line fit shown on the right insert in a log-linear representation as a function of $f_{x,y}^{3}$. This behavior is found for all the vortex structures nucleated in samples with dilute correlated disorder irrespective of the vortex density, but the force component value at which the departure from the Gaussian behavior starts increases with *B*. Data for the structures nucleated in $B_{\Phi }=5000$ G samples is again special: The PDF’s of $f_{x,y}$ follow an apparent linear evolution with $f_{x,y}^2$ on log-linear representation, irrespective of the vortex density up to 100 G (see for instance the pink data shown in Fig. 5a,b). Then, for a medium with dense correlated disorder the distributions of the force components are Gaussian as in the case of a host sample with weak and dense point disorder. This finding suggests that, in a more general perspective, Gaussian tails are expected for the PDF’s of the vortex-vortex force when pinning is in the weak limit (as is the case in the $B_{\Phi }=5000$ G samples^67^) in contrast to the algebraic tails detected in the case of dilute correlated disorder producing a strong pinning. Data on superconducting samples with strong point pins could allow to study this implication in the future.

The non-Gaussian tails observed in the PDF’s of the vortex-vortex force components originate in closely-located vortices, corresponding to the cluster-like regions observed in samples with dilute CD correlated disorder, see Fig. 1 and the bordeaux vortices in the force maps of Fig. 3. On the contrary, in the case of point and dense correlated disordered media no tendency to vortex clustering is observed and this might be at the origin of the PDF’s of the force components being well fitted by a Gaussian function in the whole force range. Indeed, a closer look to the pair distribution function of Fig. 2 for $r/a_{0} < 1$ reveals that the probability of finding vortices at very small distances $\sim 0.3-0.8\, r/a_{0}$ is larger in the case of correlated disorder than of point disorder.

## Discussion

In an attempt to explain how the spatial distribution of the particle-particle interaction force entails the different short-scale density variations in point and correlated disordered media, we examine the force distribution that would be expected from model spatial configurations. In general, the probability distribution of any of the components of the interaction force between a given pair of vortices, $p(f^{pair}_{x})$, relates to the probability density $p'(r,\theta )$ of finding one vortex at the origin and another at a position $(r, \theta )$ (polar coordinates) by the expression

2 $$\begin{aligned} p(f^{pair}_{x})=\int _0^\infty \int _0^{2\pi }p'(r, \theta )\delta (f^{pair}_{x}- {\cal{F}} (r)\cos (\theta )) r d\theta dr, \end{aligned}$$

where ${\cal{F}} (r) \propto K_{1}(r/\lambda _{\text{ab}}(T_{\text{freez}}))$ is the interaction force between any pair of vortices separated a distance *r*. Note that this pair-force is not the same as the component $f_x$ in Eq. (1), obtained as the sum of the interactions of one vortex with the rest. Nevertheless, we expect that $p(f_{x}) \approx p(f^{pair}_{x}=f_{x})$ in the limit of large $f_x$, as large forces arise from few very close neighbors. Therefore, both distributions should have tails with the same shape. While the number of vortex pairs contributing to the tails of $p(f_x)$ is small, the behavior of $p(f_x)$ at small $f_x$ is controlled by a large number pair-forces proportional to $\sim (d/a_0)^2$, with *d* roughly the size of the field-of-view. Since short-range correlations are expected for most of these finite variance contributions, we can invoke the central limit theorem to assert that $p(f_x)$ presents a Gaussian shape around $f_x=0$. Then this explains why, regardless of the type of disorder, the PDF’s of $f_{x,y}$ follow a Gaussian behavior in the low force range, see data on Figs. 4 and 5.

**Figure 6:**
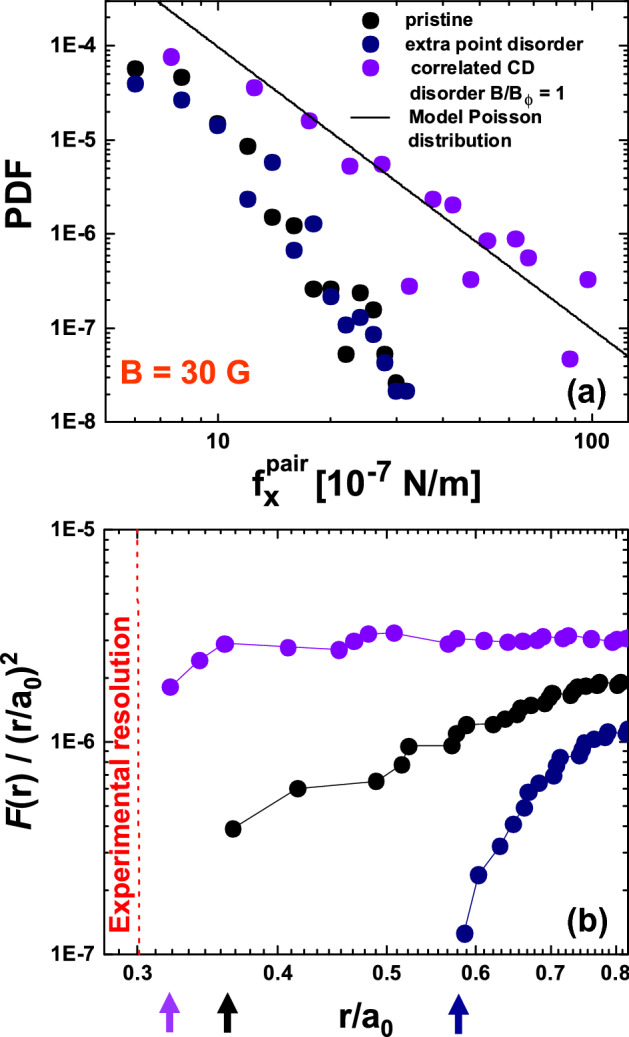
(**a**) Probability density functions (PDF’s) of the component of the interaction force between pairs of vortices, $$f^{pair}_{x}$$, for the vortex structures nucleated in samples with point (black and navy points) and dilute correlated CD (violet points) disorder with $$B_{\Phi }=30$$ G. All the data correspond to a vortex density of 30 G. The analytical $$1/f_{x}^3$$ result for a toy-model structure with a non-vanishing $$g(r) \approx F (r)/(r/a_{0})^2$$ at small distances (such as a vortex arrangement following a random Poissonian spatial distribution) is shown with a black line. (**b**) Normalized cumulative distribution function of the distance between vortices in a pair, $$ F (r)/(r/a_{0})^{2}$$, for the smallest detected $$r/a_{0}$$ values for the same vortex structures studied in panel (**a**). The threshold to resolve individual vortices with our implementation of the magnetic decoration technique, $$\sim \lambda (4.2\,\hbox {K})/a_{0}$$, is indicated with a red dashed line. Color-coded arrows located at the bottom indicate the $$r/a_{0}$$ values corresponding to the smallest detected distance between vortices in a pair in the whole field-of-view.

Let us now focus on the tails of the force distribution and consider the rather general fluid-like case of having an isotropic vortex distribution. In this particular case, $p'(r, \theta ) = 2\pi g(r)$ and integrating Eq. (2) over $\theta$ we get

3 $$\begin{aligned} p(f^{pair}_{x})=\int _0^{ {\cal{F}} ^{-1}(f^{pair}_{x})} \frac{4\pi r g(r)}{ {\cal{F}} (r)\sqrt{1-(f^{pair}_{x}/ {\cal{F}} (r))^2}}dr \end{aligned}$$

where ${\cal{F}} ^{-1}$ represents the inverse function of $\cal{F}$. Since $\cal{F}$ decreases monotonically with *r*, it is therefore invertible, and then the integration limit is uniquely defined. In order to analytically estimate the tails in the $p(f^{pair}_{x})$ distributions, we consider the infinite family of pair correlation functions that rise as $g(r) \sim r^{\alpha }$ for $r \ll a_0$, with $\alpha \ge 0$ a characteristic exponent. In addition, we consider that ${\cal{F}} ^{-1}(f) \sim 1/f$ for large *f* since ${\cal{F}} (r) \propto K_1(r/\lambda ) \sim 1/r$ for $r \sim \lambda \ll a_0$. With these assumptions, Eq. (3) can be integrated to obtain

4 $$\begin{aligned} p(f^{pair}_{x}) \propto [f^{pair}_{x}]^{-(3+\alpha )} \frac{\Gamma \left( \frac{\alpha}{2}+1\right) }{\Gamma \left( \frac{\alpha+3}{2}\right) }, \end{aligned}$$

where $\Gamma (x)$ is the Gamma function. We hence conclude that for large forces $p(f_{x}=f) \sim p(f^{pair=f}_{x}) \propto 1/|f|^{(3+\alpha )}$ (using the equivalence of the tails of the PDF’s at large *f*). In particular, for a Poissonian ideal gas-like distribution of particles we have $g(r)=1$ and then $\alpha =0$. The prediction for this case is then $p(f_{x}=f) \sim p(f^{pair}_{x}=f) \propto 1/f^{3}$. This result is however more general, as the same result holds for any isotropic particle distribution with a non-zero *g*(*r*) at the smallest observable vortex–vortex distance. Most often, for normal fluids with strong repulsive interactions, for $r \ll a_{0}$ *g*(*r*) rises slower than any power law. In this case, we can interpret that $\alpha =\infty$ effectively and then that $p(f_{x}) \sim p(f^{pair}_{x})$ decays faster than a power-law for large forces. Although these predictions are for large $f_x$ it is important to keep in mind that they should be valid if $f_x< {\cal{F}} (r_{min})$, where $r_{min}$ is a cut-off distance. This cut-off distance is given by the experimental resolution to resolve individual vortices. In magnetic decorations, $r_{min} \sim \lambda (T)$, with *T* the temperature at which the experiment is performed.

We now check the theoretical predictions described above by comparing with our experimental data of the PDF’s of $f^{pair}_{x}$ for structures nucleated at 30 G in samples with point (pristine and electron irradiated) and dilute correlated CD ($B_{\Phi }=30$ G) disorders, see Fig. 6 (a). The black curve corresponds to the analytical $1/f^3$ result found for a Poissonian toy-model structure with a non-vanishing $g(r) \approx F(r)/r^2=cte$ for $r \le a_{0}$, where $F(r)$ is the cumulative probability (see below). The figure reveals that the vortex structure nucleated in a medium with dilute correlated disorder displays a fair $1/(f^{pair}_{x})^3$ power-law decay. In contrast, structures nucleated in pristine and extra point disordered media display both a faster than power-law decay at large $f^{pair}_{x}$. These findings are in agreement with the tails of the PDF’s of the vortex–vortex interaction force components shown in Fig. 4. This confirms the assumption that the PDF’s of $f^{pair}_{x}$ and $f_{x,y}$ share the same rare events statistics.

To test further the connection between the PDF’s of the forces and the distribution of distances between vortices forming a pair (*g*(*r*)), we compute the cumulative distribution of such distance, $$ F (r)\equiv \int _0^r dr'\;2\pi r' g(r')$$, for the smallest $$r/a_{0}$$ values detected experimentally. In order to avoid spurious data binning effects for this rare events statistics, we exploit the fact that the exact $$ F (r)$$ can be directly obtained from the data. To do this we first sort all vortex-vortex distances from the smallest to the largest and use them as the horizontal coordinate. We thus obtain the exact $$ F (r)$$ for the discrete data-set by computing the order-number divided by the total number of vortex pairs. If we now model the rising $$g(r)\sim r^{\alpha }$$, we have $$ F (r)\sim r^{2+\alpha }$$. Therefore, $$ F (r)/(r/a_{0})^2 \sim (r/a_{0})^\alpha $$ gives us access to the effective exponent $$\alpha $$ which also controls the decay of the PDF’s of the interaction force. Figure  6b shows $$ F (r)/(r/a_{0})^2$$ for the 30 G vortex structures nucleated in media with weak point and dilute strong correlated disorder. In the latter case, the cumulative distribution of distances between vortices in a pair displays an almost flat behavior down to the minimum value of this magnitude detected experimentally (indicated with a violet arrow). Moreover, no tendency to a steep decrease of $$ F (r)/(r/a_{0})^2$$ is observed on decreasing $$r/a_{0}$$. This means that $$\alpha =0$$ effectively, in fair consistence with the $$1/(f_{x,y})^3$$ force distribution tails detected in structures nucleated in samples with dilute correlated disorder. On the other hand, for structures nucleated in point disordered media, $$ F (r)/(r/a_{0})^2$$ displays a faster-than-algebraic decay on decreasing $$r/a_{0}$$, with a faster decay for the sample with extra point disorder than for the pristine one. In both cases the minimum value of distance between vortices in a pair observed in the entire field-of-view is well above the experimental resolution, indicating that $$\alpha =\infty $$ effectively. The prediction for point disordered media is then a faster than algebraic decay of the force distributions, in agreement with the Gaussian-shaped PDF’s observed experimentally. The application of this analysis is only possible for systems which are dense enough to present a competition between interaction and pinning forces such that the PDF has an appreciable variance. These are also the systems for which phase transitions between glassy and liquid phases can be expected from moderate changes in the applied field and temperature. However, for sparse systems where the density can not be easily controlled, other methods, such as dynamical probes, may be used to study the force statistics.

Finally, it is worth mentioning that the prediction of Eq. (4) is rather robust since it does not require a precise knowledge on the interaction potential and on the *g*(*r*) but rather their assymptotic behaviors at short distances. Indeed, as described in the Supplementary Note 6, Eq. (4) can be generalized to a great family of systems described by $F(r)\sim 1/r^\beta$ and $g(r)\sim r^{\alpha }$ at short distances, yielding $p(f) \propto f^{-\frac{2+\alpha +\beta }{\beta }}$ for $\alpha +\beta >-2$. Note also that estimating *p*(*f*) in a given system can be used to compute the density $n_{dep}$ of particles that are near a depinning threshold or maximum pinning force $f_{dep}$, as $n_{dep} \approx p(f_{dep})\epsilon$ with $\epsilon = (f_{dep}-f)$ the force-distance to the threshold. The PDF tails can thus be thought as a susceptibility of the particle system controlled by $f_{dep}$, $\alpha$ and $\beta$.

## Conclusions

In summary, we present an alternative way on inferring the nature of the dominant disorder present in the media where elastic objects are nucleated based on the analysis of physical properties of the interacting elastic objects that can be computed from direct imaging of the structures in fields-of-view containing a statistically meaningful number of particles. We illustrate our proposal using experimental data on vortex lattices in superconducting materials as a case-study system. We analyze the statistical distribution of the disorder-induced spatially-varying particle–particle interaction force and found a behavior distinctive for strong dilute correlated as opposed to weak point-like disorder. We show that detecting non-Gaussian algebraically-decaying tails in the PDF of the components of the interaction forces acting on individual vortices is a smoking gun proof of the randomly distributed disorder, in our case dominated by dilute correlated defects acting as strong pinning centers. This result contrasts with the Gaussian PDF’s of the force components for structures nucleated in media with point or very dense correlated disorder. By considering a toy-model system we explain that the non-Gaussian tails result from inhomogeneous short-scale vortex density fluctuations associated to the tendency of clustering in some patches of the structure. Whether our method is effective to distinguish between the host media presenting strong or weak disorder in a more general perspective remains as an interesting open question for further investigations. Nevertheless, our proposal is a very promising way of inferring the nature of disorder in the host media of elastic objects from physical properties of the structures directly imaged. Its applications can be easily spanned to a wide range of soft condensed matter systems in which distinguishing the nature of disorder might be crucial for technological applications.

## Methods

The studied samples are nearly optimally-doped single crystals of $\hbox {Bi}_2\hbox {Sr}_2\hbox {CaCu}_2\hbox {O}_{8+\delta }$ from different sample growers, with natural and introduced defects distributed at random. We studied a set of roughly 40 samples grown by means of the traveling-solvent-floating-zone ^64^ and flux methods and having $T_{\text{c}} \sim 90$ K^65^. While some of these samples were kept pristine, others were exposed to different doses and types of irradiation. One underdoped sample was irradiated with electrons with an energy of 2.3 MeV and a dose of $1.7 \cdot 10^{19}$ $\hbox {e/cm}^2$ at the École Polytechnique, France. The induced damage by this irradiation resulted in extra point disorder, reduced the critical temperature of the sample down to 66 K, and raised $\lambda$ by roughly $30\%$^66^. Correlated CD disorder was generated by irradiating other pristine $\hbox {Bi}_2\hbox {Sr}_2\hbox {CaCu}_2\hbox {O}_{8+\delta }$ samples with heavy-ions at the GANIL facility in France. Some samples were irradiated with 6 GeV Pb-ions at corresponding matching fields of $\text {B}_{\Phi }=45$, 100 and 5000 G, and others with 5 GeV Xe-ions with $\text {B}_{\Phi }=30$ G. Heavy-ion irradiation produced a random poissonian distribution of CD parallel to the c-axis of the sample. In these samples there was a negligible depression of the critical temperature and no significant change in the value of $\lambda (0)$^67^.

Snapshots of the vortex structure at the surface of the sample are obtained by performing magnetic decoration experiments at 4.2 K after a field-cooling process^42^. During this process the vortex structure gets frozen at length-scales of the lattice parameter $a_0$ at a temperature $T_{\text {freez}}$ and on further cooling down 4.2 K vortices move in lengthscales of the order $\xi$, much smaller than the typical size of a vortex detected by magnetic decoration, of the order of $\lambda$. Therefore the structure imaged in such magnetic decoration experiments corresponds to the equilibrium one at $T_{\text{freez}}$. At this crossover temperature the bulk pinning dominates over the vortex-vortex repulsion and the thermal fluctuations^26,63^. Then $T_{\text{freez}}$ depends not only on the superconducting material but also on the particular pinning landscape and the magnetic induction *B*. We estimate $T_{\text{freez}}$ as of the order of the irreversibility temperature $T_{\text {irr}}$ at which bulk pinning sets in on cooling.

In order to obtain $T_{\text{irr}}(B)\sim T_{\text{freez}}$ for each particular sample, we measure the irreversibility line by means of local Hall probe magnetometry using micrometric Hall sensors with active areas of $16 \times 16$ $\mu \hbox {m}^{2}$^68^. The irreversibility temperature is taken at the onset of the non-linear magnetic response due to the growing relevance of bulk pinning on cooling. This onset is detected by measuring the vortex magnetic response to an ac ripple field superimposed to the external static magnetic field *H*, both parallel to the c-axis of the sample^68^. By applying a lock-in technique, the response of the sample at the third harmonic of the excitation field is recorded as a function of temperature and normalized as to obtain the transmittivity $\mid T_{\text{h3}} \mid$. This magnitude is zero in the normal state and starts to have a finite value on cooling at the temperature at which pinning sets in, namely $T_{\text{irr}}(B)$^53,68,69^. Measurements were typically performed with a ripple field of 1 Oe and 7.1 Hz.

## Supplementary information

Supplementary Information 1.

## Data Availability

All relevant data are available from the authors upon request.
