# Author Correction: Non-Gaussian tail in the force distribution: a hallmark of correlated disorder in the host media of elastic objects

**DOI:** 10.1038/s41598-021-99110-5

**Published:** 2021-09-27

**Authors:** Jazmín Aragón Sánchez, Gonzalo Rumi, Raúl Cortés Maldonado, Néstor René Cejas Bolecek, Joaquín Puig, Pablo Pedrazzini, Gladys Nieva, Moira I. Dolz, Marcin Konczykowski, Cornelis J. van der Beek, Alejandro B. Kolton, Yanina Fasano

**Affiliations:** 1 Centro Atómico Bariloche and Instituto Balseiro, CNEA, CONICET and Universidad Nacional de Cuyo, 8400 San Carlos de Bariloche, Argentina; 2 Universidad Nacional de San Luis and Instituto de Física Aplicada, CONICET, 5700 San Luis, Argentina; 3 Laboratoire des Solides Irradiés, CEA/DRF/IRAMIS, Ecole Polytechnique, CNRS, Institut Polytechnique de Paris, 91128 Palaiseau, France; 4 Centre de Nanosciences et de Nanotechnologies, CNRS, Université Paris-Saclay, 91120 Palaiseau, France

Correction to: *Scientific Reports* 10.1038/s41598-020-76529-w, published online 10 November 2020

The original version of this Article contained an error in the Results section, where,

“These images correspond to zooms on structures of ∼7000 vortices nucleated at 60 G in media with point (top panels) and correlated CD disorder (bottom panels).”

now reads:

“These images correspond to zooms on structures of ∼7000 vortices nucleated at 30 Gauss in media with point (top panels) and correlated CD disorder (bottom panels).”

In addition, the Discussion section contained a repeated error where the symbol “F” for force was incorrectly captured in Equations 2 and 3 and the adjacent paragraphs.

“*F*”

now reads:

 “$$\cal{F}$$”.

Furthermore, in Equation 4,

“*a*”.

now reads:

“α”.

Finally, in the Discussion section,

“The black curve corresponds to the analytical $$1/f^3$$ result found for a Poissonian toy-model structure with a non-vanishing $$g(r) \approx F(r)/r^2 = cte$$ for $$ r<a_{0}$$”

now reads:

“The black curve corresponds to the analytical $1/f^3$ result found for a Poissonian toy-model structure with a non-vanishing $g(r) \approx F(r)/r^2 = cte$ for $r<a_{0}$, where $F(r)$ is the cumulative probability (see below).”

The original Article has been corrected.

