# Supplementary material for: Non-Gaussian tail in the force distribution: a hallmark of correlated disorder in the host media of elastic objects
Source: Sci Rep. 2020 Nov 10;10:19452. doi: 10.1038/s41598-020-76529-w (PMC7655960; doi:10.1038/s41598-020-76529-w)
Supplement: Supplementary file 1 — Supplementary Information 1. [file 41598_2020_76529_MOESM1_ESM.pdf]

Supplementary material to: The non-Gaussian tail in the force distribution is a hallmark of correlated disorder in the host media of elastic objects

Jazmín Aragón Sánchez,[1] Gonzalo Rumi,[1] Raúl Cortés Maldonado,[1] Néstor René Cejas Bolecek,[1] Joaquín Puig,[1] Pablo Pedrazzini,[1] Gladys Nieva,[1] Moira I. Dolz,[2] Marcin Konczykowski,[3] Cornelis J. van der Beek,[4] Alejandro B. Kolton,[1] and Yanina Fasano*[1]

[1]*Centro Atómico Bariloche and Instituto Balseiro, CNEA,*
CONICET and Universidad Nacional de Cuyo, 8400 San Carlos de Bariloche, Argentina
[2]*Universidad Nacional de San Luis and Instituto de Física Aplicada, CONICET, 5700 San Luis, Argentina.*
[3]*Laboratoire des Solides Irradiés, CEA/DRF/IRAMIS, Ecole Polytechnique,*
CNRS, Institut Polytechnique de Paris, 91128 Palaiseau, France
[4]*Centre de Nanosciences et de Nanotechnologies, CNRS,*
Université Paris-Sud, Université Paris-Saclay, 91120 Palaiseau, France.

Supplementary Note 1

In the case of a superconductor with correlated pinning centers generated on cleaving the samples, strong fluctuations of the first-neighbor distances has been proposed as the fingerprint of a gel-like phase of vortices.[47] In the opposite scale of long-range vortex density fluctuations, some of us reported on the vortex structure nucleated in the cuprate superconductor $Bi_2Sr_2CaCu_2O_{8+\delta}$ with point and correlated pinning centers being hyperuniform at the sample surface.[46] A very recent study reveals disordered vortex structures observed in several pnictides and conventional superconductors seem also to be hyperuniform.[60] Hyperuniformity is a topological property of a state of matter characterized by strongly-reduced long-wavelength density fluctuations entailing an algebraically decaying structure factor for small wave-vectors. According to our study,[46] vortex structures nucleated in samples with a random distribution of point pins are theoretically expected to present hyperuniform structural properties. However, in the particular case of correlated pinning centers generated by columnar defecs (CD), no hyperuniform vortex structures are theoretically expected. Even though, the experimentally measured decay of the structure factor on decreasing q in these samples is consistent with the structure being hyperuniform. We interpreted this apparent discrepancy between theory and experiment as a consequence of the viscous freezing of the vortex structure when field-cooling from the vortex liquid phase towards the glassy vortex phase. In order to be reliable, these studies on long-range vortex density fluctuations require the direct imaging of individual vortex positions in extended fields-of-view. Then, applying these hyperuniformity studies in order to infer information on the nature of the pinning centers present in superconducting samples relies on having extended and high-resolution snapshots with thousand of vortices or more, which is sometimes technically challenging.

Supplementary Note 2

In order to calculate the local particle-particle interaction force for the structures nucleated in every studied medium, we have to consider the penetration depth at the freezing temperature $T_{\rm freez}$ at which disorder in the medium hinders the meandering of vortices in length-scales larger than a_0. This temperature is of the order of that at which pinning sets in, namely the irreversibility temperature $T_{\rm irr}$.[53,63] We measure the field depen-

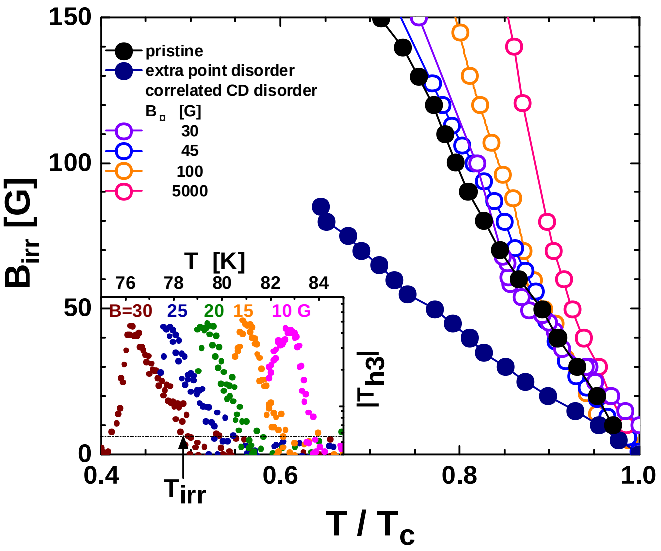

Figure 1. Irreversibility lines $B_{\rm irr}(T)$ for the $Bi_2Sr_2CaCu_2O_{8+\delta}$ samples studied in this work with point disorder (pristine and electron-irradiated) and correlated CD disorder (heavy-ion-irradiated). The matching fields B_Φ for the samples with correlated disorder are indicated. Inset: Temperature-evolution of the modulus of the third-harmonic signal $\mid T_{\rm h3} \mid$ obtained from ac magnetization measurements. Data for various fields are shown for the illustrative example of $B_\Phi = 30\,$G. The irreversibility temperature $T_{\rm irr}$ is determined from the onset of non-linearities in the magnetic response, namely at the point indicated with an arrow where $\mid T_{\rm h3} \mid$ overcomes the experimental noise level (horizontal line) on cooling.

dence of $T_{\rm irr}$ by means of local Hall magnetometry techniques.[68] At a given field, $T_{\rm irr}$ is estimated from the onset of non-linearities in the magnetic response, namely at the temperature where $\mid T_{\rm h3} \mid$ overcomes the experimental noise level on cooling, see arrow in the inset to Fig. 1. Then data are inverted to obtain the $B_{\rm irr}$ as a function of temperature shown in Fig. 1. This figure shows the irreversibility line $B_{\rm irr}(T)$ for all samples studied in this work.

For samples in its pristine form and those with correlated disorder, the slope of $B_{\rm irr}$ is quite similar in a $T/T_{\rm c}$ scale (and also in a T scale since $T_{\rm c}$ is similar for both sets of samples). The case of samples with extra point disorder generated by electron irradiation is different: the slope of $B_{\rm irr}$ is reduced concomitantly with a lowering of $T_{\rm c} = 66\,$K.

The irreversibility lines for each sample are considered to obtain the value of $\lambda(T_{\rm freez} \approx T_{\rm irr})$ that enters into the vortex-vortex interaction force. We assume the temperature dependence $\lambda(T_{\rm freez}) = \lambda_{\rm ab}(0)/\sqrt{1-(T_{\rm freez}/T_{\rm c})^4}$ and consider $\lambda(0) = 180\,$nm for samples with correlated disorder and pristine,[53,67] and $\lambda_{\rm ab}(0) = 230\,$nm for samples with extra point disorder.[66] Extra point disorder introduced by electron irradiation produces a depletion in the superfluid density of the material and then $\lambda(0)$ increases. [66]

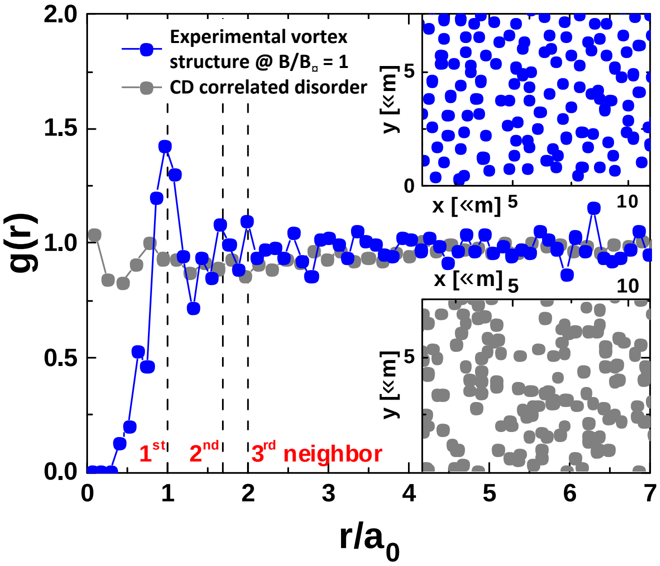

Figure 2. Pair correlation functions for the experimental vortex structure nucleated at $B/B_\Phi = 1$ (blue curve) in the $Bi_2Sr_2CaCu_2O_{8+\delta}$ sample with correlated disorder with $B_\Phi = 30\,$G, and for a random Poissonian distribution of CD correlated disorder with the same density than that of vortices (gray curve). Top inset: vortex positions observed experimentally. Bottom inset: positions of CD following a poissonian distribution with a density equal to the matching field of $B_\Phi = 30\,$G.

Supplementary Note 3

The density of vortices in Figs. 1 (c) and (d) of the main text is equal to the matching field $B_\Phi = 30\,$G of the correlated CD pinning distribution. Then, in this case there is globally the same number of vortices and pinning sites. However, this does not mean that every vortex is located on top of a CD. This can be studied by comparing the pair correlation function for the experimentally observed vortex structure with that of a structure with the same vortex density but spatially arranged following a poissonian distribution as that of the CD landscape.[21] The insets to Fig. 2 show a zoom-in of the vortex structure observed experimentally (top panel) and of the distribution of correlated pinning centers following a poissonian distribution (bottom panel) for both structures with the same global density. This comparison shows that the vortex structure nucleated in samples with correlated disorder for $B/B_\Phi = 1$ is disordered, but particles do not follow a poissonian distribution in the sample: The $g(r)$ of the experimental structure presents clear peaks at distances corresponding to the first, second and third neighbors of a hexagonal lattice, whereas that of CD poissonian distribution fluctuates around 1 at all r/a_0. Then, the experimental structure does not mimic the correlated pinning distribution and moreover, presents a hyperuniform disorder as we reported previously.[46] This implies that, at some particular locations, since the spatial distribution of CD is poissonian, some defects are closer than a_0 and locating vortices on these particular pins is energetically unfavorable. This lack of mimicking of the pinning landscape by the particles will also hold in the case of poissonian distributed point pinning centers, given the gain in pinning energy is even smaller in this latter case as to balance the loss of elastic energy. Therefore, studying the $g(r)$ of elastic structures at densities commensurate with that of correlated disorder ($B/B_\Phi = 1$) does not seem a promising way for ascertaining whether disorder in the media is correlated or point-like.

Supplementary Note 4

In the fields of view shown in Fig. 1 of the main text, no topological defects are observed in the samples with point disorder (pristine and electron irradiated) at $B = 30\,$G. In contrast, there are plenty of non-sixfold coordinated vortices in samples with correlated disorder (heavy-ion irradiated): 54 % at the same magnetic induction. We studied larger fields-of-view containing up to 15000 vortices at every B in order to have statistics on the field-dependence of the density of topological defects, $\rho_{\rm def}$, see results in Fig. 3(a). For point disorder, at low magnetic inductions the structure is policrystalline, but on increasing field the size of crystallites enhances and for fields $B > 15\,$G becomes single-crystalline. This produces a decrease in $\rho_{\rm def}$ on increasing B. In pristine samples $\rho_{\rm def}$ decreases from 48 % at 4 G to 1.1 % at 28 G. In the case

of the electron irradiated sample, $\rho_{\rm def}$ dropped from 33 % to 0.3 % at the same B.[45] In contrast, when nucleated in a sample with correlated disorder, the vortex structure is amorphous at low fields and presents small crystallites with less than 150 vortices on increasing field. This results in large values of $\rho_{\rm def} > 40\,\%$ up to 100 G. The inset to Fig. 3 (a) shows $\rho_{\rm def}$ as a function of B/B_Φ for the vortex structures nucleated in samples with CD that we study here and published data obtained in other samples for comparison.[34] For densities $B/B_\Phi < 2$ the vortex structure is amorphous and $\rho_{\rm def} \sim 50\,\%$. On further increasing B/B_Φ the vortex structure becomes policrystalline and $\rho_{\rm def}$ decreases systematically down to $\sim 13\,\%$ at $B/B_\Phi \sim 16$. This figure shows that at high vortex densities $\rho_{\rm def}$ is quantitatively different for point than for correlated disorder, but at low densities $\rho_{\rm def} \sim 50\,\%$ in both cases.

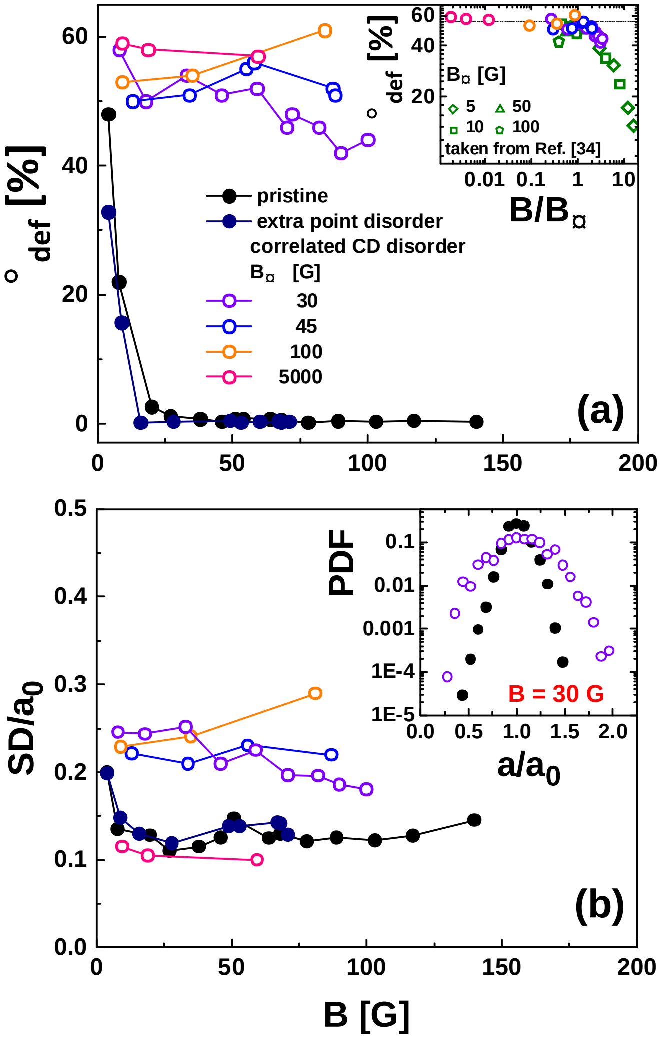

Figure 3. (a) Density of topological defects as a function of vortex density B for vortex structures nucleated in $Bi_2Sr_2CaCu_2O_{8+\delta}$ samples with point and correlated disorder. Samples with point disorder are pristine and irradiated with electrons, whereas correlated disorder is produced by CD generated by heavy-ion irradiation resulting in different defect density quantified by the matching field B_Φ. Inset: density of defects in the vortex structure nucleated in samples with correlated disorder as a function of B/B_Φ for the samples studied here and data for Pb-irradiated samples from the literature. (b) Standard deviation of the first-neighbors distances, SD, normalized by the mean lattice spacing, a_0, for the samples studied here. Inset: Probability density function of the normalized first-neighbors distance a/a_0 for a vortex density of 30 G nucleated in pristine and heavy-ion irradiated ($B_\Phi = 30$ G) samples.

Another magnitude that can be quantified from real-space imaging of elastic structures nucleated in disordered media is the density fluctuation at first-neighbors distance, obtained from the spatially inhomogeneous distribution of first-neighbor distances a. In structures nucleated in a media with point disorder, a rather uniform density of vortices is observed. In contrast, a tendency to vortex clustering is usually observed in structures nucleated in samples with strong and dilute correlated disorder. This difference in particle density-fluctuations at first-neighbor distance can be quantified by computing the standard deviation of the spatial distribution of first-neighbor distances, SD. Examples of the probability density function of first-neighbor distances, a, normalized by its mean value a_0, are shown in the inset to Fig. 3 (b). This figure shows data of structures nucleated at 30 G in samples with point and correlated CD disorder, the distribution of the latter being noticeably wider than that of the former.

The main panel of Fig. 3 (b) depicts the evolution of the normalized magnitude SD/a_0 as a function of vortex density B for all the studied cases. Curves for structures nucleated in samples with point disorder, irrespective of its magnitude (pristine or extra disorder generated by electron-irradiation), are packed at low SD/a_0 values of roughly 0.2 at very low densities and 0.13 at intermediate vortex densities. Data for structures nucleated in samples with dilute correlated disorder, $B_\Phi = 30 - 100$ G in our case, are also packed around a larger value of SD/a_0 varying roughly between 0.2-0.3 in the whole studied vortex density. This discrepancy in the vortex density fluctuations at first-neighbor distance is presumably due to difference in the density and magnitude of pinning centers, being dense and weak for samples with point disorder and diluted and strong for samples with CD. In the latter case, the gain in pinning energy when trying to mimic the CD landscape can account for the larger fluctuations in a. The structures nucleated at $B \ll B_\Phi$ for a dense distribution of pins with $B_\Phi = 5000$ G seem to follow a different phenomenology: The values of SD/a_0 measured in this case are close to those of samples with point disorder. This can be explained considering that the density of pins, even they are correlated and strong, is between 80 to 400 times larger than the particle density and a vortex can profit from a pinning center with no need of generating large a fluctuations. Then, the magnitude and field-evolution of SD/a_0 is not an unambiguous

indicative of disorder being point or correlated in nature, but can give a hint on the media presenting dilute correlated disorder if its magnitude is larger than 20 % for intermediate vortex densities. Nevertheless, this criteria is not very qualitative nor sufficiently clear to apply.

Supplementary Note 5

As mentioned in the main text, the pair correlation function $g(r)$ is an angular-averaged probability that gives information on short- and intermediate-distance vortex density variations. For instance, in the extreme cases of an ideal gas, $g(r)$ has a value of 1 independently of r/a_0, whereas for a perfect lattice presents delta functions at distances corresponding to first neighbors, second neighbors, and so on. The particular geometry of the lattice gives the r/a_0 values at which these peaks are observed, see for instance the inset of Fig. **??** (a) for the case of a perfect hexagonal structure. Disorder in the host sample shortens the positional order of the structure and then induce a widening of these delta functions.

In the case of samples with point disorder, at the lower vortex density of 8 G, $g(r)$ is quite similar for structures nucleated in pristine and electron-irradiated samples. However, on increasing field, sharper peaks are developed in the case of pristine samples, see Fig. **??** (b). On enhancing field there is a systematic increase of the number of peaks observed in $g(r)$ for structures nucleated in both types of point-disordered media, in accordance with vortex-vortex interaction becoming more relevant with B. For a given vortex density, the $g(r)$ for structures nucleated in samples with point disorder presents several sharp peaks in contrast to a lesser number of peaks detected for structures nucleated in a medium with correlated disorder. This washing out of the peaks produced by correlated disorder suggests in this medium pinning dominates over vortex-vortex interaction and produces a substantial enhancement of the displacement of particles with respect to the sites of a perfect hexagonal lattice even at distances as short as $r/a_0 \sim 2$. However, using the number of peaks detected in $g(r)$ and its sharpness seems not a categorical criterion to determine whether disorder in the medium is dominated by point or correlated pins. For instance, for the 68 G structure nucleated in a sample with correlated disorder ($B_\Phi = 30\,\mathrm{G}$), three peaks in $g(r)$ are clearly distinguished, a phenomenology also observed in samples with point pins at low vortex densities.

Supplementary Note 6

Here we generalize the prediction for the power-law tails in the PDF's of the particle-particle interaction force components to the case where the repulsive force is vaguely known, except for its asymptotic shape at short distances, assumed to be of the form

$$F(r) \sim r^{-\beta}, \tag{1}$$

with $\beta \geq 0$ a characteristic exponent. For instance, $\beta = 13$ for the Lennard-Jones potential, $\beta = 1$ for straight parallel interacting vortices or line charges in electrostatics, $\beta = 2$ for point electrostatic charges, for Van der Walls forces between macroscopic objects (e.g. some colloids) or for the Yukawa potential, $\beta = 0$ for non-divergent potentials such as the Morse potential and also in the case of straight superconducting vortices but at distances smaller than the coherence length (a rare situation which is beyond the magnetic decoration resolution).

The non-trivial competition between interactions and pinning will produce configurations characterized by a $g(r)$ that, in the small-distance limit, we can assume to have the following generic form

$$g(r) \sim r^{\alpha}, \tag{2}$$

where α is a characteristic exponent describing the vanishing (or the divergence) of $g(r)$ at short length-scales. It is interesting to note that, at equilibrium and in the absence of disorder, $g(r) \sim \exp[-w_2(r)/k_\mathrm{B}T]$, where $w_2(r)$ is the potential of the mean force. Since in the dilute limit $w_2(r)$ becomes the true interaction potential, at small distances and in the particular case for $\beta \neq 1$, the pair correlation function $g(r) \sim \exp[-Ar^{-\beta+1}/k_BT]$ (with A a constant). This may be represented effectively by $\alpha \to \infty$ if $\beta > 1$ or $\alpha = 0$ if $\beta < 1$. For $\beta = 1$ a pure power-law $g(r) \sim \exp[-A\log(1/r)/k_BT] \sim r^{A/k_BT}$ is obtained and so $\alpha = A/k_BT$. However, in the presence of pinning forces the link of $g(r)$ with the interaction force is not direct, and even the thermal equilibrium assumption should be checked. Nevertheless, in the non dilute limit and in a static, though not necessarily thermal equilibrium condition, we can write, as in the main text, the PDF for one component f of the pair-interaction force,

$$p(f) = \int_0^{F^{-1}(f)} dr\, \frac{4\pi r g(r)}{F(r)\sqrt{1-(f/F(r))^2}} dr \tag{3}$$

where F^{-1} represents the inverse function of F. Since from Eq.1 F decreases monotonically with r at short lenght scales, it is therefore invertible, and then the integration limit is uniquely defined. Using Eqs. 1 and 2 we hence get,

$$\begin{aligned} p(f) &= \int_0^{1/f^{1/\beta}} dr\, \frac{4\pi r^{1+\alpha+\beta}}{\sqrt{1-(f^{1/\beta}r)^\beta}} dr \\ &= f^{-\frac{2+\alpha+\beta}{\beta}} I \end{aligned} \tag{4}$$

where

$$I = \int_0^1 du \frac{u^{1+\alpha+\beta}}{\sqrt{1-u^{2\beta}}}. \tag{5}$$

is a dimensionless finite number if $\alpha + \beta > -2$,

$$I = \begin{cases} \frac{\sqrt{\pi}\Gamma\left[\frac{2+\alpha+\beta}{2\beta}\right]}{(2+\alpha)\Gamma\left[\frac{2+\alpha}{2\beta}\right]}, & \text{if } \beta > 0 \\ (\beta+2)^{-1}, & \text{if } \beta = 0 \end{cases} \quad (6)$$

In either case, we get

$$p(f) \propto f^{-\frac{2+\alpha+\beta}{\beta}}, \quad (7)$$

generalizing the expression for $\alpha = 0$ and $\beta = 1$ obtained in the main text. When $\beta = 0$ and $\alpha > -2$, Eq. 7 should be interpreted as a faster than power-law decay. It is interesting to note that $-2 < \alpha < 0$ is allowed, corresponding to an isotropic fractal aggregate of particles, with $\alpha = d_f - d$, d the space dimension and d_f the fractal dimension of the aggregate.

It is interesting to separately discuss the $\alpha = 0$ case corresponding to a Poisson distribution at small scales. This distribution of particles is expected in the presence of randomly-distributed strong pinning centers which are able to pin nearby particles in spite of their repulsion (as in the case of our data in samples with columnar defects). In this case, $p(f) \sim f^{-(2+\beta)/\beta}$ and for large β it converges to an universal $p(f) \sim 1/f$ law. This result approximates the cases of particles interacting with rapidly diverging potentials such as Lennard-Jones ($\beta = 13$), where $p(f) \sim f^{-15/13}$, or other hard particles.

The above calculation shows that a precise knowledge of the interaction force is not needed in order to apply our method to distinguish between point and correlated disorder, but only its behavior at short distances. The same is true for $g(r)$, but its behavior at short distances is difficult to infer, except in the case of strong pins. In this latter case estimating the tails of $p(f)$ for a particle system allows to infer the number of particle pairs near the single pin depinning force f_{dep}, as $\delta N \approx N p(f_{dep})\epsilon$, where $\epsilon = f_{dep} - f \ll f_{dep}$ is the force-distance to the threshold. In this sense $p(f_{dep}) \sim f_{dep}^{-(2+\alpha+\beta)/\beta}$ can be think of as a susceptibility of the pinned system that depends on the strength f_{dep} and the exponents characterizing $g(r)$ and the pair interaction force $F(r)$ at short distances.

[1] Moretti, P., Miguel, M.C., Zaiser, M. & Zapperi, S. , Depinning transition of dislocation assemblies: Pileups and low-angle grain boundaries, Phys. Rev. B **69**, 214103 (2004).

[2] Ponson, L. & Pindra, N., Crack propagation through disordered materials as a depinning transition: A critical test of the theory, Phys. Rev. E **95** , 053004 (2017).

[3] Ji, H. & Robbins, M. O., Transition from compact to self-similar growth in disordered systems: Fluid invasion and magnetic-domain growth, Phys. Rev. A **44**, 2538 (1991).

[4] Lemerle, S., Ferré, J., Chappert, C., Mathet, V., Giamarchi, T. & Le Doussal, P., Domain Wall Creep in an Ising Ultrathin Magnetic Film, Phys. Rev. Lett. **80**, 849 (1998).

[5] Brazovskii, S. & Nattermann, T., Pinning and sliding of driven elastic systems: from domain walls to charge density waves, Adv. Phys. **53**, 177 (2004).

[6] Gruner, G., The dynamics of charge-density waves, Rev. Mod. Phys. **60**, 1129 (1988).

[7] Man, W., Florescu, M., Williamson, E. P., He, Y., Hashemizad, S. R., Leung, B. Y. C., Liner, D. R., Torquato, S., Chaikin, P. M., & Steinhardt, P. J., Isotropic band gaps and freeform waveguides observed in hyperuniform disordered photonic solids, Proceed. Nat. Acad. Sci. **110**, 15886 (2013).

[8] Kurita, R. & Weeks, E. R., Incompressibility of polydisperse random-close-packed colloidal particles, Phys. Rev. E **84**, 030401(R) (2011).

[9] Kulikova, D. P., Pyatako, A. P., Nikolaeva, E. P., Sergeev, A. S., Kosykh, T. B., Pyatakova, Z. A., Nikolaev, A. V. & Zvezdin, A. K. Nucleation of Magnetic Bubble Domains in Iron Garnet Films by Means of an Electric Probe, JETP Lett. **104** 197 (2016).

[10] Murray, C. A., Sprenger, W. O. & Wenk, R. A., Comparison of melting in three and two dimensions: Microscopy of colloidal spheres, Phys. Rev. B **42**, 688 (1990).

[11] Urbach, J. S., Madison, R. C. & Markert, J.T., Interface Depinning, Self-Organized Criticality, and the Barkhausen Effect, Phys. Rev. Lett. **75**, 276 (1995).

[12] Blatter, G., Feigel'man, M. V., Geshkenbein, V. B., Larkin, A. I. & Vinokur, V. M., Vortices in high-temperature superconductors, Rev. Mod. Phys. **66**, 1125 (1994).

[13] Giamarchi, T. & Le Doussal, P., Elastic theory of flux lattices in the presence of weak disorder, Phys. Rev. B **52**, 1242 (1995).

[14] Nattermann, T. & Scheidl, S., Vortex-glass phases in type-II superconductors, Adv. Phys. **49**, 607 (2000).

[15] Le Doussal, P. & Wiese, K., Driven particle in a random landscape: disorder correlator, avalanche distribution and extreme value statistics of records, Phys. Rev. E **79**, 051105 (2009).

[16] Guyonnet, J., Agoritsas, E., Bustingorry, S., Giamarchi, T. & Paruch, P., Multiscaling Analysis of Ferroelectric Domain Wall Roughness, Phys. Rev. Lett. **109**, 147601 (2012).

[17] Fasano, Y., De Seta, M., Menghini, M. Pastoriza, H. & de la Cruz, F., Commensurability and stability in nonperiodic systems, Proc. Natl. Acad. Sci. **102**, 3898 (2005).

[18] Wu, Y. L., Derks, D., van Blaaderen, A. & Imhof, A., Melting and crystallization of colloidal hard-sphere suspensions under shear, Proc. Natl. Acad. Sci. **106**, 10564 (2009).

[19] Dreyfus, R., Xu, Y., Still, T., Hough, L. A., Yodh, A. G. & Torquato, S., Diagnosing hyperuniformity in two-dimensional, disordered, jammed packings of soft spheres, Phys. Rev. E **91**, 012302 (2015).

[20] Weijs, J. H., Jeanneret, R., Dreyfus, R. & Bartolo, D., Emergent Hyperuniformity in Periodically Driven Emulsions, Phys. Rev. Lett. **115**, 108301 (2015).

[21] Leghissa, M., Gurevich, L. A., Kraus, M., Saemann-

Ischenko, G. & Vinnikov, L. Y., Observation of a disordered vortex state in $Bi_2Sr_2CaCu_2O_{8+\delta}$ single crystals containing columnar defects, Phys. Rev. B **48**, 1341 (1993).
[22] Dai, H., Yoon, S., Liu, J., Budhani, R. C. & Lieber, C. M., Simultaneous Observation of Columnar Defects and Magnetic Flux Lines in High-Temperature $Bi_2Sr_2CaCu_2O_8$ Superconductors, Science **265**, 1552 (1994).
[23] Harada, K., Kamimura, O., Kasai, H., Matsuda, T., Tonomura, A. & Moshchalkov, V. V., Direct Observation of Vortex Dynamics in Superconducting Films with Regular Arrays of Defects, Science **274**, 1167 (1996).
[24] Bezryadin, A., Ovchinnikov, Y. N. & Pannetier, B., Nucleation of vortices inside open and blind microholes, Phys. Rev. B **53**, 8553 (1996).
[25] Troyanovski, A. M., Aarts, J. & Kes, P. H., Collective and plastic vortex motion in superconductors at high flux densities, Nature **399**, 665 (1999).
[26] Fasano, Y., Herbsommer, J. A., de la Cruz, F., Pardo, F., Gammel, P. L., Bucher, E. & Bishop, D.J., Observation of periodic vortex pinning induced by Bitter decoration, Phys. Rev. B **60**, 15047 (1999).
[27] Fasano, Y., Herbsommer, J. A. & de la Cruz, F., Superficial Periodic Pinning Induced by Bitter Decoration Applied to the Study of Vortex Structure Nucleation and Growth, Phys. Stat. Sol. (b) **215**, 563 (1999).
[28] Fasano, Y., Menghini, M., De la Cruz, F. & Nieva, G., Weak interaction and matching conditions for replicas of vortex lattices, Phys. Rev. B **62**, 15183 (2000).
[29] Grigorenko, A. N., Howells, G. D., Bending, S. J., Bekaert, J., Van Bael, M. J., Van Look, L., Moshchalkov, V. V., Bruynseraede, Y., Borghs, G., Kaya, I. I. & Stradling, R. A., Direct imaging of commensurate vortex structures in ordered antidot arrays, Phys. Rev. B **63**, 052504 (2001).
[30] Surdeanu, R., Wijngaarden, R. J., Griessen, R., Einfeld, J. & Wördenweber, R., Visualization of novel flux dynamics in $YBa_2Cu_3O_{7-x}$ thin films with antidots, Europhys. Lett. **54**, 682 (2001).
[31] Silevitch, D. M., Reich, D. H., Chien, C. L., Field, S. B. & Shtrikman, H., Imaging and magnetotransport in superconductor/magnetic dot arrays, Journal App. Phys. **89**, 7478 (2001).
[32] Field, S. B., James, S. S., Barentine, J., Metlushko, V., Crabtree, G., Shtrikman, H., Ilic, B. & Brueck, S. R. J., Vortex Configurations, Matching, and Domain Structure in Large Arrays of Artificial Pinning Centers, Phys. Rev. Lett. **88**, 067003 (2002).
[33] Menghini, M., Fasano, Y. & de la Cruz, F., Critical current and topology of the supercooled vortex state in $NbSe_2$, Phys. Rev. B **65**, 064510 (2002).
[34] Menghini, M., Fasano, Y., de la Cruz, F., Banerjee, S. S., Myasoedov, Y., Zeldov, E., van der Beek, C. J., Konczykowski, M. & Tamegai, T., First-Order Phase Transition from the Vortex Liquid to an Amorphous Solid, Phys. Rev. Lett. **90**, 147001 (2003).
[35] Van Bael, M. J., Lange, M., Raedts, S., Moshchalkov, V. V., Grigorenko, A. N., & Bending, S. J. , Local visualization of asymmetric flux pinning by magnetic dots with perpendicular magnetization, Phys. Rev. B **68**, 014509 (2003).
[36] Fasano, Y., De Seta,M., Menghini, M., Pastoriza, H., & de la Cruz, F., Imaging the structure of the interface between symmetries interconnected by a discontinuous transition, Solid State Comm. **128**, 51 (2003).
[37] Veauvy, C., Hasselbach, K., & Mailly, D. , Micro-SQUID microscopy of vortices in a perforated superconducting Al film, Phys. Rev. B **70**, 214513 (2004).
[38] Karapetrov, G., Fedor, J., Iavarone, M., Rosenmann, D. & Kwok, W. K., Direct Observation of Geometrical Phase Transitions in Mesoscopic Superconductors by Scanning Tunneling Microscopy, Phys. Rev. Lett. **95**, 167002 (2005).
[39] Björnsson, P. G., Maeno, Y., Huber, M. E. & Moler, K. A., Scanning magnetic imaging of Sr_2RuO_4, Phys. Rev. B **72**, 012504 (2005).
[40] Yurchenko, V. V., Wördenweber, R., Galperin, Y. M., Shantsev, D. V., Vestgå rden, J. I., & Johansen, T. H., Magneto-optical imaging of magnetic flux patterns in superconducting films with antidotes, Phys. C, **437-438**, 357 (2006).
[41] Fischer, Ø., Kugler, M., Maggio-Aprile, I., Berthod, C. & Renner, Ch., Scanning tunneling spectroscopy of high-temperature superconductors, Rev. Mod. Phys. **79**, 353 (2007).
[42] Fasano, Y. & Menghini, M., Magnetic-decoration imaging of structural transitions induced in vortex matter, Supercond. Sci. Tech. **21**, 023001 (2008).
[43] Petrovic, A. P., Fasano, Y., Lortz, R., Senatore, C., Demuer, A., Antunes, A. B., Paré, A., Salloum, D., Gougeon, P., Potel, M. & Fischer, Ø., Real-Space Vortex Glass Imaging and the Vortex Phase Diagram of $SnMo_6S_8$, Phys. Rev. Lett. **103**, 257001 (2009).
[44] Suderow, H., Guillamón, I., Rodrigo, J. G. & Vieira, S., Imaging superconducting vortex cores and lattices with a scanning tunneling microscope, Supercond. Sci. Tech. **27**, 063001 (2014).
[45] Aragón Sánchez, J., Cortés Maldonado, R., Cejas Bolecek, N. R., Rumi, G., Pedrazzini, P., Dolz, M. I., Nieva, G., van der Beek, C. J., Konczykowski, M., Dewhurst, Ch. D., Cubbit, R., Kolton, A., Pautrat, A. & Fasano, Y., Unveiling the vortex glass phase in the surface and volume of a type-II superconductor, Comm. Phys. **2**, 143 (2019).
[46] Rumi, G., Aragón Sánchez, J., Elías, F., Cortés Maldonado, R., Puig, J., Cejas Bolecek, N. R., Nieva, G., Konczykowski, M., Fasano, Y. & Kolton, A. B., Hyperuniform vortex patterns at the surface of type-II superconductors, Phys. Rev. Res. **1**, 033057 (2019).
[47] Llorens, J. B., Embon, L., Correa, A., González, J. D., Herrera, E., Guillamón, I., Luccas, R. F., Azpeitia, J., Mompeán, F. J., García-Hernndez, M., Munuera, C., Aragón Sánchez, J., Fasano, Y., Miloševic, M. V., Suderow, H. & Anahory, Y., Observation of a gel of quantum vortices in a superconductor at very low magnetic fields, Phys. Rev. Res. **2**, 013329 (2020).
[48] Demirdis, S., van der Beek, C. J., Fasano, Y., Cejas Bolecek, N. R., Pastoriza, H., Colson, D. & Rullier-Albenque, F., Strong pinning and vortex energy distributions in single-crystalline $Ba(Fe_{1-x}Co_x)_2As_2$, Phys. Rev. B **84**, 094517 (2011).
[49] Yang,H., Shen, B., Wang, Z., Shan, L., Ren, C. & Wen, H.-H., Vortex images on $Ba_{1-x}K_xFe_2As_2$ observed directly by magnetic force microscopy, Phys. Rev. B **85**, 014524 (2012).
[50] van der Beek, C. J., Demirdis, S., Konczykowski, M., Fasano, Y., Cejas Bolecek, N. R., Pastoriza, H., Colson, D. & Rullier-Albenque, F., Vortex pinning: A probe for nanoscale disorder in iron-based superconductors, Phys. B **407**, 1746 (2012).
[51] Demirdis, S., Fasano, Y., Kasahara, S., Terashima, T., Shibauchi, T., Matsuda, Y., Konczykowski, M., Pastoriza, H. & van der Beek, C. J., Disorder, critical cur-

rents, and vortex pinning energies in isovalently substituted $BaFe_2(As_{1-x}P_x)_2$, Phys. Rev. B **87**, 094506 (2013).

[52] Yagil, A., Lamhot, Y., Almoalem, A., Kasahara, S., Watashige, T., Shibauchi, T., Matsuda, Y. & Auslaender, O. M., Diamagnetic vortex barrier stripes in underdoped $BaFe_2(As_{1-x}P_x)_2$, Phys. Rev. B **94**, 064510 (2016).

[53] Cejas Bolecek, N. R., Kolton, A. B., Konczykowski, M., Pastoriza, H., Domínguez, D. & Fasano, Y., Vortex matter freezing in $Bi_2Sr_2CaCu_2O_8$ samples with a very dense distribution of columnar defects, Phys. Rev. B **93**, 054505 (2016).

[54] Aragón Sánchez, J., Cortés Maldonado, R., Dolz, M. I., CejasBolecek, N. R., van der Beek, C. J., Konczykowski, M. & Fasano, Y., Direct visualization of local interaction forces in $Bi_2Sr_2CaCu_2O_{8+d}$ vortex matter, Materials Today: Proceedings **14**, 34 (2019).

[55] Fisher, D. S., Fisher, M. P. A. & Huse, D. A., Thermal fluctuations, quenched disorder, phase transitions, and transport in type-II superconductors, Phys. Rev. **43**, 130 (1991).

[56] Nelson, D. R. & Vinokur, V. M., Boson localization and correlated pinning of superconducting vortex arrays, Phys. Rev. B **48**, 13060 (1993).

[57] Giamarchi, T. & Le Doussal, P., Elastic theory of flux lattices in the presence of weak disorder, Phys. Rev. B **55**, 6577 (1997).

[58] Civale, L., Vortex pinning and creep in high-temperature superconductors with columnar defects, Supercond. Sci. Technol. **10**, A11 (1997).

[59] Fedirko, V. A., Kasatkin, A. L. & Polyakov, S. V., Vortex Escape from Columnar Defect in a Current-Loaded Superconductor, J. Low Temp. Phys **192**, 359 (2018).

[60] Llorens, J. B. , Guillamón, I., García-Serrano, I., Córdoba, R., Sesé, J., De Teresa, J. M., Ibarra, M. R., Vieira, S., Ortuño, M. & H. Suderow, Disordered hyperuniformity in superconducting vortex lattices, Phys. Rev. Res. **2**, 033133 (2020).

[61] Kalisky, B., Kirtley, J. R., Analytis, J. G., Chu, J.-H., Fisher, I. R., & Moler, K. A., Behavior of vortices near twin boundaries in underdoped $Ba(Fe_{1-x}Co_x)_2As_2$, Phys. Rev. B **83**, 064511 (2011).

[62] Oral, A., & Bending, S. J., Real-time scanning Hall probe microscopy, Appl. Phys. Lett. **69**, 1324 (1996).

[63] Pardo, F., Mackenzie, A. P., de la Cruz, F. & Guimpel, J., Effect of the reversibility region on the low-temperature vortex structure imaged by Bitter magnetic decoration, Phys. Rev. B **55**, 14610 (1997).

[64] Li, T. W., Kes, P. H., Hien, N. T., Franse, J. J. M. & Menovsky, A. A., Growth of $Bi_2Sr_2CaCu_2O_{8+x}$ single crystals at different oxygen ambient pressures, J. Cryst. Growth 135, 481 (1994).

[65] Correa, V. F., Kaul, E. E. & Nieva, G., Overdoping effects in $Bi_2Sr_2CaCu_2O_{8+x}$: From electromagnetic to Josephson interlayer coupling, Phys. Rev. B **63**, 172505 (2001).

[66] Konczykowski, M., van der Beek, C. J., Koshelev, A. E., Mosser, V., Li, M. & Kes, P. H., Vortex matter in $Bi_2Sr_2CaCu_2O_{8+x}$ with pointlike disorder, J. Phys. Conf. Ser. **150**, 052119 (2009).

[67] van der Beek, C. J., Konczykowski, M., Drost, R. J., Kes, P. H., Chikumoto, N. & Bouffard, S., Entropy, vortex interactions, and the phase diagram of heavy-ion-irradiated $Bi_2Sr_2CaCu_2O_{8+x}$, Phys. Rev. B **61**, 4259 (2000).

[68] Dolz, M. I., Fasano, Y., Pastoriza, H., Mosser, V., Li, M. & Konczykowski, M., Latent heat and nonlinear vortex liquid in the vicinity of the first-order phase transition in layered high-T_c superconductors, Phys. Rev. B **90** (14), 144507.

[69] Dolz, M. I. , Pedrazzini, P., Fasano, Y., Pastoriza, H., & Konczykowski, M., Effect of quenched disorder in the entropy-jump at the first-order vortex phase transition of $Bi_2Sr_2CaCu_2O_{8+d}$, J. Low Temp. Phys., **179**, 28 (2015).
